# Nothing about us without us: considerations for ensuring rights‐affirming index case testing for young people

**DOI:** 10.1002/jia2.25595

**Published:** 2020-08-11

**Authors:** Lauren E Parmley, Joseph G Rosen, Oğuzhan Nuh, Manuel Venegas, Aaron Sunday, Ali İ Nergiz, Aron Thiim, the AIDS 2020 Youth Force

**Affiliations:** ^1^ ICAP at Columbia University New York NY USA; ^2^ Department of International Health Johns Hopkins Bloomberg School of Public Health Baltimore MD USA; ^3^ Social Policy Gender Identity, and Sexual Orientation Studies Association Istanbul Turkey; ^4^ defeatHIV Community Advisory Board Fred Hutch Cancer Research Center Seattle WA USA; ^5^ African Network of Adolescents and Young Persons Development (ANAYD) Kaduna Kaduna State Nigeria; ^6^ Association of Positive Youth Living with HIV in Nigeria (APYIN) Abuja Nigeria; ^7^ Cerrahpasa Medical School Istanbul University‐Cerrahpasa Istanbul Turkey; ^8^ Program RISE Justice Resource Institute Framingham MA USA

**Keywords:** youth, adolescents, index case testing, partner notification services, young people living with HIV, young key populations

Index case testing (ICT)—HIV testing among exposed sexual, injection and biological (i.e. children) contacts of people living with HIV, often those who are newly diagnosed or virally unsuppressed—is an effective approach to optimize HIV testing by increasing positivity yields (or the proportion of people testing HIV‐positive among those tested). ICT may also optimize linkage to and uptake of HIV prevention services, including pre‐exposure prophylaxis and voluntary medical male circumcision, among contacts who do not regularly access health services and who test HIV‐negative.

In 2019, the U.S. President’s Emergency Plan for AIDS Relief (PEPFAR) prioritized optimization of HIV testing, including scaling up ICT and partner notification services (PNS), to enhance HIV case identification. PNS, under the umbrella of ICT, can be assisted or made through passive referrals. Assisted PNS can include provider support to patients to disclose their HIV status to partners or provider‐initiated anonymous disclosure to partners and HIV testing [1]. In contrast, passive PNS includes patients disclosing their HIV status to their partners on their own and encouraging them to seek HIV testing [1]. Across HIV testing modalities, ICT, including PNS, produced the highest yield and identified the second largest number of HIV cases across PEPFAR‐supported programmes in 2019 [2].

The scale‐up of ICT, while effective, has been met with concern from advocates, who have outlined potential human rights concerns, including rights to informed consent, exposure to violence and criminalization of HIV exposure and/or transmission [1, 3, 4]. Equally, advocates have raised concerns about PEPFAR establishing country‐specific targets for ICT and their resulting impacts on service quality [3]. Concerns and guidance on appropriate delivery of ICT have been broad and silent on special considerations for youth populations. This Viewpoint reflects concerted, collaborative efforts among young scholars and advocates globally to address this gap in guidance and articulate considerations to guide implementation of ICT for youth.

In January 2020, following reports of violence as a result of ICT and assisted PNS and denial of HIV services to patients refusing to provide contacts to providers, all PEPFAR programmes were directed to halt ICT for key populations (KP), including female sex workers, people who inject drugs and men who have sex with men [4, 5]. PEPFAR has since lifted this guidance, but mandates that all facilities implementing ICT meet minimum standards in compliance with the World Health Organization’s Guidelines on HIV Self‐Testing and Partner Notification [6], though formal PEPFAR guidance has not been publicly disseminated. As PEPFAR and national HIV programmes continue to develop guidance on monitoring mechanisms to assess facilities’ capacity to implement and safely deliver ICT, considerations to ensure confidential, voluntary and rights‐affirming ICT for youth, including those belonging to KP, must be prioritized. The AIDS 2020 Youth Force has outlined key considerations to guide implementation of ICT for and with young people:

1. *Patient‐provider power dynamics*. Power dynamics between youth patients and providers/counsellors may have greater imbalance than those between adult patients and providers/counsellors [7, 8, 9]. Providers/counsellors can represent positions of authority, both as individuals who manage patients’ HIV and other health‐related care as well as elders or peer role models in their community. When unacknowledged, these imbalances can result in coercive interactions between providers/counsellors and their younger patients. Youth may feel pressured or obliged, for example to accept ICT when offered by providers/counsellors due to this power imbalance and without full comprehension of potential adverse events, especially when interactions with providers are rushed or brief.
2. *Intimate partner violence (IPV)*. Youth have various types of romantic and transactional sexual partners, including peers as well as older partners (e.g. sugar daddies, blessers) [10, 11, 12, 13]. PNS for youth must consider and be differentiated according to sexual partners, as IPV risk may vary across partner type. Youth, especially young women in heterosexual partnerships, may face severe repercussions, including IPV due to HIV status disclosure to sexual partners [14, 15], and those in partnerships of dependence may be unable to escape. PNS can be particularly risky in circumstances where young people report concurrent sexual partners, each of whom will be traced and informed of a potential exposure to HIV from a sexual partner, which may disclose to a contact that their partner has been unfaithful and increase risk of IPV. Moreover, each concurrent sexual partner presents individual IPV risk for the index patient.
3. *Unintended disclosure of sexual and social identities*. For young KP, ICT may have economic, legal and social repercussions, including loss of clients/wages, relationship dissolution, IPV and/or gender‐based violence, stigma and discrimination, arrest, isolation from peers and families, and other undue incrimination [16]. In addition to putting young KP at risk, ICT may also expose sexual and/or social identities of their clients, sexual partners, and/or drug‐injecting partners, which may result in similar repercussions for contacts. Beyond the inclusion of minimum standards for implementing ICT with KP, considerations for young KP must be incorporated into guidance.
4. *Unintended disclosure of HIV status/sexual activity*. ICT may involve inadvertent disclosure of the HIV status and/or sexual activities of youth to family members and/or peers. For example home visits by counsellors/community health workers may inadvertently disclose sexual activities of youth to family members/parents. Similarly, ICT as part of HIV testing campaigns at schools or universities may have unintended consequences of disclosure to peers. Partner elicitation can also disclose the HIV status and/or sexual activities of sexual contacts to providers/counsellors, individuals who may also be contacts’ community members, without their consent. Moreover, perceived or actual breaches of patient–provider confidentiality may further disincentivize or alienate youth from accessing HIV services in their communities. Steps to ensure confidentiality of ICT among youth must be outlined in guidance and monitoring mechanisms, and confidentiality upheld.

While ICT and PNS are vital strategies to strengthen HIV case identification, these approaches must be thoroughly interrogated considering suboptimal implementation fidelity. The AIDS 2020 Youth Force advises PEPFAR and national HIV programmes to consult and meaningfully engage youth living with and affected by HIV as minimum standards and monitoring mechanisms for ICT are developed to ensure acceptability, safe delivery and uptake among youth.

## COMPETING INTERESTS

The authors declare no competing interests.

## AUTHORS’ CONTRIBUTIONS

LP and JGR drafted viewpoint. ON, MV, AS, AIN and AT reviewed viewpoint. All authors approved the contents of this Viewpoint.

## AUTHORS’ INFORMATION

The AIDS 2020 Youth Force aims to ensure inclusive and meaningful youth engagement in the AIDS 2020 conference. All authors are Co‐Chairs of the AIDS 2020 Youth Force Working Groups or members of the AIDS 2020 Youth Force. LP serves as the Co‐Chair of the Advocacy Working Group for the AIDS 2020 Youth Force. ON, MV and AS serve as IAS Youth Ambassadors. AIN serves as the Co‐Chair of the Youth Pre‐Conference Working Group for the AIDS 2020 Youth Force. AT serves as the Co‐Chair of the Global Village Programming Working Group for the AIDS 2020 Youth Force.

## ABBREVIATIONS

ICT, Index case testing; IPV, Intimate partner violence; KP, Key populations; PEPFAR, U.S. President’s Emergency Plan for AIDS Relief; PNS, Partner notification services.
